# Implications of Central Obesity-Related Variants in *LYPLAL1, NRXN3, MSRA,* and *TFAP2B* on Quantitative Metabolic Traits in Adult Danes

**DOI:** 10.1371/journal.pone.0020640

**Published:** 2011-06-02

**Authors:** Dorthe S. Bille, Karina Banasik, Johanne M. Justesen, Camilla H. Sandholt, Annelli Sandbæk, Torsten Lauritzen, Torben Jørgensen, Daniel R. Witte, Jens-Christian Holm, Torben Hansen, Oluf Pedersen

**Affiliations:** 1 The Novo Nordisk Foundation Center for Basic Metabolic Research, Section of Metabolic Genetics, Faculty of Health Sciences, University of Copenhagen, Copenhagen, Denmark; 2 The Children's Obesity Clinic, Department of Paediatrics, Holbæk University Hospital, Holbæk, Denmark; 3 Faculty of Health Sciences, University of Copenhagen, Copenhagen, Denmark; 4 Novo Nordisk A/S, Medical and Science, Development Projects, Bagsværd, Denmark; 5 Department of General Practice, University of Aarhus, Aarhus, Denmark; 6 Research Centre for Prevention and Health, Glostrup University Hospital, Glostrup, Denmark; 7 Steno Diabetes Center, Gentofte, Denmark; 8 Faculty of Health Sciences, University of Southern Denmark, Odense, Denmark; 9 Hagedorn Research Institute, Gentofte, Denmark; 10 Faculty of Health Sciences, University of Aarhus, Aarhus, Denmark; Aarhus University, Denmark

## Abstract

**Background:**

Two meta-analyses of genome-wide association studies (GWAS) have suggested that four variants: rs2605100 in lysophospholipase-like 1 *(LYPLAL1),* rs10146997 in neuroxin 3 (*NRXN3),* rs545854 in methionine sulfoxide reductase A *(MSRA),* and rs987237 in transcription factor activating enhancer-binding protein 2 beta (*TFAP2B)* associate with measures of central obesity.

To elucidate potential underlying phenotypes we aimed to investigate whether these variants associated with: 1) quantitative metabolic traits, 2) anthropometric measures (waist circumference (WC), waist-hip ratio, and BMI), or 3) type 2 diabetes, and central and general overweight and obesity.

**Methodology/Principal Findings:**

The four variants were genotyped in Danish individuals using KASPar®. Quantitative metabolic traits were examined in a population-based sample *(n* = 6,038) and WC and BMI were furthermore analyzed in a combined study sample (*n* = 13,507). Case-control studies of diabetes and adiposity included 15,326 individuals. The major G-allele of *LYPLAL1* rs2605100 associated with increased fasting serum triglyceride concentrations (per allele effect (β) = 3%(1;5(95%CI)), *p*_additive_ = 2.7×10^−3^), an association driven by the male gender (*p*_interaction_ = 0.02). The same allele associated with increased fasting serum insulin concentrations (β = 3%(1;5), *p*_additive_ = 2.5×10^−3^) and increased insulin resistance (HOMA-IR) (β = 4%(1;6), *p*_additive_ = 1.5×10^−3^). The minor G-allele of rs10146997 in *NRXN3* associated with increased WC among women (β = 0.55cm (0.20;0.89), *p*_additive_ = 1.7×10^−3^, *p*_interaction_ = 1.0×10^−3^), but showed no associations with obesity related metabolic traits. The *MSRA* rs545854 and *TFAP2B* rs987237 showed nominal associations with central obesity; however, no underlying metabolic phenotypes became obvious, when investigating quantitative metabolic traits. None of the variants influenced the prevalence of type 2 diabetes.

**Conclusion/Significance:**

We demonstrate that several of the central obesity-associated variants in *LYPLAL1, NRXN3, MSRA*, and *TFAP2B* associate with metabolic and anthropometric traits in Danish adults. However, analyses were made without adjusting for multiple testing, and further studies are needed to confirm the putative role of *LYPLAL1*, *NRXN3, MSRA*, and *TFAP2B* in the pathophysiology of obesity.

## Introduction

Obesity is a major health problem with increasing prevalence in Western societies, and obesity, as well as the often associated insulin resistance (IR), are important risk factors for type 2 diabetes, cardiovascular diseases, hypertension, and several other chronic diseases. To provide new insight into the pathophysiology of obesity, genome-wide association studies (GWAS) have in the last few years been performed with elaborating results [1], [2], [3], [4], [5], [6], [7] identifying variants associated with body mass index (BMI) – an indirect measure of general adiposity. Following, two meta-analyses demonstrated four loci to associate with measures of central adiposity (waist circumference (WC) and waist–hip-ratio (WHR)): 1) a meta-analysis of 16 GWAS (*n* = 38,580) with follow-up studies in a maximum of 118,691 individuals of European origin, identified variants near lysophospholipase-like 1 *(LYPLAL1),* methionine sulfoxide reductase A *(MSRA),* and transcription factor activating enhancer-binding protein 2 beta *(TFAP2B),* to strongly associate with measures of central adiposity using information about adult WC and WHR [8], and 2) a large-scale meta-analysis of GWAS from the CHARGE Consortium involving 70,014 Caucasian individuals identified neuroxin 3 (*NRXN3)* as a novel locus for central adiposity [9].

Lysophospholipase-like 1 protein, encoded by *LYPLAL1,* is thought to act as a triglyceride lipase and reported to be up-regulated in subcutaneous adipose tissue of obese individuals [10]. In the meta-analysis of 16 GWAS, the major G-allele of rs2605100 in *LYPLAL1* associated with increased WHR (*p* = 2.6×10^−8^) only among women [8]. *NRXN3* encodes the neuroxines protein family that functions in the vertebrate nervous system as cell adhesion molecules and receptors. In patients with schizophrenia, *NRXN3* has been demonstrated to associate with alcohol dependence and the degree of nicotine dependence [11], [12]. The minor G-allele of rs10146997 in *NRXN3* associated with WC (*p* = 6.4×10^−7^) and BMI (*p* = 7.4×10^−6^) in the CHARGE GWAS; however, the association with WC attenuated (*p* = 0.32), when adjusted for BMI [9]. *MSRA* encodes an antioxidant enzyme that repairs proteins inactivated by oxidative damage, but the biological connections between the *MSRA* locus and adiposity are unclear [8]. In the meta-analysis of 16 GWAS, a strong association was found with the minor G-allele of rs545854 in *MSRA* and central obesity measured by WC (*p* = 8.9×10^−9^) (mixed-gender analysis) [8]. The association diminished (*p* = 0.11), when adjusted for BMI.

The binding-protein encoded by *TFAP2B* is expressed in adipose tissue [13]. Studies have shown an association between *TFAP2B* and type 2 diabetes, and over-expression of *TFAP2B* in adipocytes leads to IR and accumulation of triglycerides inside the adipocytes [13], [14], [15]. In a study of 1,176 adolescents, Nordquist *et al.* found that men carrying the 4 repeat allele of intron 2 polymorphism (intronic variable number tandem repeat) of *TFAP2B* had higher insulin sensitivity and central obesity (measured by skin folds) [16]. Another study, comprising 81 individuals suggested that *TFAP2B* might be a novel candidate gene for development of the metabolic syndrome, as it was shown to regulate the expression of various adipokines [17]. In the meta-analysis of 16 GWAS, the minor G-allele of rs987237 in *TFAP2B* associated significantly with central obesity measured by WC (*p* = 1.9×10^−11^) and general obesity measured by BMI (*p* = 7.0×10^−12^ (stage 2 alone)) in the mixed-gender analysis [8]. Although these genes are considered obvious candidates for obesity, the causal variants and their role in the pathogenesis of obesity remain to be elucidated.

The aim of the present study was to investigate central obesity-associated variants in *LYPLAL1, NRXN3, MSRA*, and *TFAP2B* for associations with quantitative metabolic traits in a population-based sample of 6,038 adult Danes (The Inter99 study sample). In addition, we examined associations between these variants and anthropometric measures (WC, WHR, and BMI) in a larger group of individuals. Also, we conducted large case-control studies involving a total of 15,326 Danes, investigating associations between these variants and type 2 diabetes, as well as both central and general overweight and obesity. Since three of the variants (*LYPLAL1, MSRA*, and *TFAP2B*) in previous studies have been investigated using sex-stratified analyses, we performed all analyses stratified according to sex.

## Results

In the Inter99 population, the major G-allele of rs2605100 in *LYPLAL1* associated with fasting serum triglyceride concentrations (per allele effect(β) = 3%, ((95% confidence interval(CI))1;5), *p*_additive (add)_ = 2.7×10^−3^), fasting serum insulin concentrations (β = 3% (1;5), *p*_add_ = 2.5×10^−3^), and homeostasis model assessment-insulin resistance (HOMA-IR) (β = 4% (1;6), *p*_add_ = 1.5×10^−3^) (table 1). When stratifying according to sex the association with fasting serum triglycerides was restricted to men (β = 6% (3;9), *p*_add_ = 2.4×10^−4^) (table 1). Indeed, the interaction analysis revealed an interaction between sex and genotype for triglyceride levels (*p_int_* = 0.02) (Table S2). Furthermore, we found a borderline association between rs2605100 and decreased BMI (β = −0.18 kg/m^2^ (−0.36;−1×10^−3^), *p*_add_ = 0.05), and decreased WC (β = −0.08 cm (−0.29;0.13), *p*_add_ = 0.04) (table 1). However, the association with WC diminished, when adjusted for BMI (*p*_add_ = 0.47) (Table S2). The combined quantitative trait (QT) analysis showed associations with decreased WC (β = −0.48 cm (−0.81;0.16), *p*_add_ = 0.004) and BMI (β = −0.16 kg/m^2^ (−0.28;−0.04), *p*_add_ = 0.01), but the associations diminished, when data were adjusted for BMI (*p*_add_ = 0.12) and WC (*p*_add_ = 0.87), respectively (Table S3 and Table S4). In the case-control study the variant associated with central obesity, measured by WC (Odds Ratio(OR)_add_ = 0.92 (0.86–0.98(95% CI)), *p*_add_ = 0.01) (table 2), and the association strengthened, when WC was adjusted for BMI (*p*_add_ = 0.004) (Table S3). No other significant associations were found for central or general obesity (table 2).

**Table 1:** Variants showing statistical significant associations with quantitative metabolic traits (*n* = 6,038).

	*Quantitative metabolic traits*						
*Variant*	*BMI (kg/m2)*	*WC (cm)*	*WHR*	*Fasting serum triglyceride*	*Fasting serum insulin*	*Fasting plasma glucose*	*Insulin resistance, HOMA-IR*
***LYPLAL1 rs2605100***							
All	−0.18 (−0.36;−1×10^−3^),0.05	−0.08 (−0.29;0.13),0.04		3% (1;5),2.7×10^−3^	3% (1;5),2.5×10^−3^		4% (1;6),1.5×10^−3^
Men				6% (3;9),2.4×10^−4^			
Women					4% (1;6),0.01	1% (0;1),0.04	4% (1;7),6.0×10^−3^
***NRXN3 rs10146997***							
Women		0.55 (0.20;0.89),0.02	4×10^−3^ (2×10^−4^;0.01),0.02				
***MSRA rs545854***							
Men					−4% (−8;−1),0.02		−5% (−9;−1),0.02

The table includes effect sizes and p-values (β (95% CI), p-value) for significant associations only.

Values of fasting serum triglycerides, serum insulin, plasma glucose, and HOMA-IR were logarithmically transformed prior to statistical analyses, and their effect sizes (β) are presented as the increase/decrease in percent. *P*-values were calculated assuming an additive model (*p*_add_). BMI, waist circumference and waist-hip ratio were adjusted for age and sex.

**Table 2:** Variants showing statistical significant associations with BMI and/or WC in the case-control analyses (*n* = 15,326).

	*BMI*	*WC*
	*OR (95%CI), p-value*	*OR (95%CI), p-value*
***LYPLAL1 rs2605100***		
All		0.92(0.86–0.98), 0.004
Men		0.89(0.82–0.97), 0.0004
***NRXN3 rs10146997***		
All	1.02 (0.94–1.11), 0.04	
Women	1.04 (0.93–1.17), 0.04	
***MSRA rs545854***		
All		1.08(1.00–1.18), 0.02
Women		1.09(0.96–1.23), 0.04
***TFAP2B rs987237***		
Men		1.01 (0.91–1.13), 0.01
Women		1.17 (1.03–1.32), 0.001

The effect is the odds ratio (OR) presented as the increase/decrease and 95% confidence interval (CI). Effect and *p*-values shown are for an additive genetic model (*p*_add_) and are adjusted for age, sex, and diabetes treatment for the obese cases. BMI, body mass index; WC, waist circumference.

The minor G-allele rs10146997 of *NRXN3* associated with increased WC among women (β = 0.55 cm (0.20;0.89), *p*_add_ = 1.7×10^−3^) (table 2) in the Inter99, which was underpinned by the interaction analysis (*p*_int_ = 1.0×10^−3^) (Table S5). The significance level attenuated, when adjusted for sex and age only (Table S5). This association was not found in the larger combined QT analysis (Table S3). Neither were we able to show any associations with other obesity-related metabolic phenotypes. In the case-control analyses the variant showed no association with general adiposity, measured by BMI (OR_add_ = 1.02 (0.94–1.11), *p*_add_ = 0.66) (Table S4). No interaction between sex and genotype was found in these analyses.

The rs545854 minor G-allele in *MSRA* associated with decreased fasting serum insulin concentrations (β = −4% (−8;−1), *p*_add_ = 0.02), and HOMA-IR (β = −5% (−9;−1), *p*_add_ = 0.02) among men (table 1). However, the interaction analysis did not show any interaction between sex and genotype for the two traits (*p_int_* = 0.08) (Table S6). The variant was not associated with other quantitative metabolic or anthropometric traits (Table S3, S4, and S6). When comparing the genotype distribution between lean and obese individuals a borderline association between rs545854 and central obesity was observed (OR_add_ = 1.08 (1.00–1.18), *p*_add_ = 0.05) (table 2), and when adjusted for BMI, this association was strengthened (*p*_add_ = 0.02) (Table S3).

The minor G-allele rs987237 in *TFAP2B* did not associate with any metabolic traits in the QT analysis (Table S3, S4, and S7). However, the variant was borderline associated with central obesity (OR_add_ = 1.08 (1.0–1.17), *p*_add_ = 0.06) in the case-control analysis (Table S3). This borderline association became nominal significant, when stratified to women (OR_add_ = 1.17 (1.03–1.32), *p*_add_ = 0.01; *p*_int_ = 0.04). When adjusted for BMI, the association became stronger among women (*p*_add_ = 0.001) and it also became evident among men (*p*_add_ = 0.01) (Table S3).

No heterogeneity between the study groups was observed for the investigated variants, except for *TFAP2B* rs987237 in the combined QT analysis in relation to general obesity (BMI) (Fixed effect, I^2^: 78%[41%–92%], *p* = 0.003) (Table S4).

None of the variants associated with central and general overweight, or type 2 diabetes in the case-control analyses (data not shown).

## Discussion

In this study we found several associations with quantitative metabolic and anthropometric traits for the central obesity-associated variants in *LYPLAL1, NRXN3, MSRA*, and *TFAP2B.*

As a major finding the G-allele (risk-allele) of rs2605100 in *LYPLAL1* associated with elevated concentrations of fasting serum triglycerides and fasting serum insulin and with estimates of central obesity. In a recent meta-analysis of 32 GWAS for WHR, a variant in *LYPLAL1* (rs4846567) in high linkage disequilibrium (r^2^ = 0.64, D' = 0.84, HapMap CEU population) with rs2605100 achieved genome-wide significance, and the subsequent association analyses with related metabolic traits in 77,167 participants (follow-up comprising up to 113,636 subjects) showed that this variant associated with increased levels of serum triglycerides (*p = *0.018), fasting insulin (*p = *1.1×10^−5^), and HOMA-IR (*p = *9.8×10^−6^) [18]. These previously reported findings strengthen the present observations in our somewhat smaller study population. Moreover, we additionally investigated sex-specific effects of rs2605100 and found that the association with fasting serum triglycerides was restricted to men. This association has not previously been reported.

Our findings for rs2605100 in *LYPLAL1* are in line with previous observations that a higher fat-mass causes increased lipogenesis, resulting in higher levels of circulating triglycerides and IR [19], [20], [21]. In mice, it has been demonstrated that insulin inhibits the breakdown of fat in adipose tissue by inhibiting the intracellular triglyceride lipase in the beta-cell [10], [21]. The elevated triglyceride concentrations may result from an increased expression of the lipase gene, which then facilitates higher triglyceride lipase activity in the adipose tissue. Furthermore, obesity is associated with an increased number and/or size of adipose cells. These cells respond more poorly to insulin, as they have impaired insulin signaling, resulting in increased activity of the lipoprotein lipases, including the triglyceride lipase. The increased lipase activity and increased mass of adipose tissue lead to an increase in circulating FFAs, which is a major contributor to the development of IR [22]. Thus, this underlying insulin resistant phenotype might add to explain the associations found between the *LYPLAL1* variant and obesity.

The minor G-allele rs10146997 in *NRXN3* associated with increased WC among women, but we found no clear association with BMI as reported in a recent meta-analysis of 249,796 individuals [23]. Neither did we observe an association between rs10146997 and fasting serum insulin levels nor IR (HOMA-IR) as reported for rs10150332 in *NRXN3* (r^2^ = 1.00, D' = 1.00, HapMap CEU population) in the meta-analysis [23]. The reason for these inconsistencies may be 1) lack of statistical power in our study due to the relatively low minor allele frequency, or 2) the association observed in the meta-analysis may reflect a random correlation between IR and BMI.

In this study, the *MSRA* rs545854 minor G-allele showed associations with decreased fasting levels of serum insulin and decreased HOMA-IR among men, but no association with obesity was evident. The latter finding is consistent with the recent meta-analyses that failed to demonstrate genome-wide significant evidence of associations between this variant and BMI or WHR [18], [23]. Also, analyses of rs987237 in *TFAP2B* showed borderline association with central obesity among women. The GWAS showed no evidence of sex-specific associations. However, *TFAP2B* was confirmed as an obesity locus in the recent meta-analysis, where this variant associated with BMI at a genome-wide significant level [23].

It is well known that substantial gender-specific differences in fat distribution exist [18]. These have been shown to reflect genetic influences i.e. on WC, and hip circumference, and for WHR the genetic variance is almost twice as large in women than in men [24]. The underlying molecular mechanisms are not clearly understood. In this study, the observed effect sizes did not provide sufficient statistical power to account for the gender stratification. Therefore, we cannot exclude that the observed gender-specific results reflect spurious findings. Further studies are needed to investigate a putative influence of gender on the associations observed for these variants.

Whether variation in the examined four genes is causal in relation to obesity remain unclear. *NRXN3* is expressed in the brain, as many of the other recently discovered obesity loci, and variants in *NRXN3* may provide changes of the brain function and behavior [11]. Eating disorders have been ascribed to the hypothalamic part of the brain [25], and recently, *NRXN3* has been investigated in relation to addiction of alcohol and smoking [11], [12]. *MSRA* is thought to be involved in the oxidative damage in cells but the molecular mechanisms are not yet elucidated. Another candidate gene located near *MSRA*, the TRF1-interacting ankyrin-related ADP-ribose polymerase (*TNKS*), has been proposed as the causal gene for the association with central obesity [8]. Previously, *TFAP2B* has been investigated for associations with several phenotypes correlated to diabetes and obesity [13], [14], [15], [16], [17], and has been proposed to be a new gene for the metabolic syndrome. We showed an association between central obesity and rs987237 *TFAP2B* minor G-allele, however, we were not able to determine the underlying phenotype for this association in the quantitative trait analysis of obesity-related measures, nor did we show any association with type 2 diabetes.

In conclusion, we found that several of the central obesity-associated variants in *LYPLAL1, NRXN3, MSRA*, and *TFAP2B* associated with metabolic and anthropometric traits among adult Danes. However, analyses were made without adjusting for multiple testing, and further studies are needed to elucidate the involvement of *LYPLAL1*, *NRXN3, MSRA*, and *TFAP2B* in the pathophysiology of obesity.

## Materials and Methods

The studies were approved by the appropriate Regional Ethics Committees, and were in accordance with the principles of the Helsinki Declaration.

### Study samples

This study comprises 15,326 Danish individuals ascertained from four different study groups (Table S1): 1) the Inter99 cohort, which is a population-based, randomized, non-pharmacological intervention study of middle-aged individuals for the prevention of ischemic heart disease (*n* = 6,162 individuals, aged 30–61 years) from the Research Centre for Prevention and Health in Glostrup, Copenhagen (ClinicalTrials.gov ID-no.: NCT00289237) [26], 2) a cohort comprising individuals with type 2 diabetes (*n = *1,695) sampled at Steno Diabetes Center (SDC), 3) a randomized, population-based group of unrelated middle-aged individuals (*n* = 730) examined at SDC, Copenhagen, and 4) The Danish ADDITION screening cohort (*n = *6,739), which is part of the Anglo-Danish-Dutch study of Intensive Treatment in People with Screen-detected Diabetes in Primary Care (ClinicalTrials.gov ID-no.: NCT00237548) [27].

A standandardized oral glucose tolerance test was undertaken in volunteers of Inter99. In this cohort QT analyses were performed in treatment-naive individuals involving individuals with normal glucose tolerance, impaired fasting glucose and impaired glucose tolerance. The exact numbers are given in tables. Patients with known type 2 diabetes (*n* = 124) were excluded from the QT analyses.

To increase statistical power, QT analyses for BMI, WC, and WHR were additionally performed including individuals from all study populations with the respective phenotypes available. Again, individuals with known type 2 diabetes were excluded. The combined study sample comprised 13,507 individuals.

The case-control studies of type 2 diabetes included all unrelated type 2 diabetic case patients and all glucose-tolerant control individuals from study group 1–4. All control individuals had normal fasting glycaemia and were glucose tolerant following an oral glucose tolerance test. Individuals from study group 4 with BMI<25 kg/m^2^ were excluded. Diabetes, impaired fasting glucose, and impaired glucose tolerance were defined in accordance with World Health Organization 1999 criteria [28].

In the case-control studies for overweight and obesity, central obesity was estimated using WC and general obesity was estimated using BMI. Overweight was defined as BMI≥25 kg/m^2^ and <30 kg/m^2^. Obesity was defined as BMI≥30 kg/m^2^. A lean control individual was defined as BMI<25 kg/m^2^. For WC the definition of overweight and obesity was sex-specific. For women overweight was defined as WC≥80 cm and <88 cm, and obesity as WC≥88 cm. The definitions for men were a WC≥94 cm and <102 cm for overweight, and a WC≥102 cm for obesity. A lean control individual was defined as WC<80 cm, for women, and WC<94 cm for men.

All participants were Danes by self-report. Before participation informed written consent was obtained from all subjects.

### Anthropometrical and biochemical measurements

The anthropometrical measurements of weight, height, WC, and hip were performed in light indoor clothes without shoes. BMI was calculated as weight in kg divided by height in m^2^. WC was measured midway between the iliac crest and the lower costal margin with the participants in standing position. Hip was measured at the level of trochante major. WC and hip were calculated in cm. The methods used to obtain biochemical measurements have been described previously [26], [27]. HOMA-IR was calculated as: (fasting plasma glucose (mmol/l) × fasting serum insulin (pmol/l))/22.5 [29].

### Genotyping

The rs2605100 in *LYPLAL1,* rs10146997 in *NRXN3,* rs545854 (former rs7826222) in *MSRA,* and rs987237 in *TFAP2B* were genotyped using KASPar® with success rates >97% and error rates  =  0%. The risk-allele frequencies were 70% (rs2605100 G-allele in *LYPLAL1*), 21% (rs10146997 G-allele in *NRXN3*), 15% (rs545854 G-allele in *MSRA*), and 17% (rs987237 G-allele in *TFAP2B*), which are in accordance with HapMap, and obeyed Hardy-Weinberg equilibrium (*p*>0.05).

### Statistical analyses

The four variants were investigated for associations with quantitative metabolic traits. The quantitative variables were tested for differences between genotyped groups using linear regression, assuming an additive model. All analyses were adjusted for sex, age, and BMI, when appropriate. BMI was additionally adjusted for WC. *P*-values given in parentheses were only adjusted for age and sex. Values of serum triglyceride, serum insulin, HOMA-IR, and plasma glucose had non-normally distributed residuals and were logarithmically transformed prior to statistical analyses. Their effect sizes (β) are presented as an increase/decrease in percent with 95% CI. Values without transformation (BMI, WC, and WHR) are given as actual values with 95% CI. The case-control studies were analyzed using logistic regression, assuming an additive model. Here we included type 2 diabetes patients, why these analyses were adjusted for sex, age and diabetes treatment, and with or without BMI or WC, respectively. Heterogeneity was assessed with the generic inverse variance meta-analysis method (R package: meta), which describes the proportion of variation in the effects that is attributable to genuine differences across the study groups rather than to random error.

To investigate whether the effect of the alleles differed between genders, we included an interaction term between sex and the genotype of interest in the linear model. In this model, we assumed an additive effect for the genotypes and sex as a binary vector. All statistical analyses were performed using R version 2.9.2. *P*-values were not adjusted for multiple hypothesis testing and *p*<0.05 was considered nominally significant.

Statistical power calculations in the case-control analyses were done using CaTS, power calculations for large genetic association studies, available at http://www.sph.umich.edu/csg/abecasis/cats/. Depending on the type of analysis, as well as the risk allele frequency (RAF) of the analyzed variant, we had between 72% and 100% power to detect genetic effects in this study (*p*<0.05) (Table S2). The lowest and the highest RAF of the examined SNPs were 15% and 70%, respectively. Using the population-based Inter99 cohort as reference, the prevalence of central overweight and obesity in the Danish population were estimated to 23% and 22%, respectively, the prevalence of general overweight and obesity in the Danish population were estimated to 39% and 17%, respectively, and the prevalence of type 2 diabetes in the examined Danish population was 5%. Our power calculations estimated a statistical power of 99% and 100% to detect associations with central overweight and obesity, respectively, for a variant with a RAF of 15% with a relative risk of 1.15 (Table S2). The statistical power estimates for quantitative traits were estimated in R using 1,000 simulations, *n* = 6,162, and with a significance threshold of 0.05 (Table S2).

## Supporting Information

Table S1 **Characteristics for individuals included in the analyses stratified according to study group.** Data are means ± standard deviation. SDC, Steno diabetes center. WC, waist circumference.
(DOCX)

Table S2 **Quantitative metabolic traits in 5,769 treatment-naïve middle-aged Danes from the population-based Inter99 cohort according to** ***LYPLAL1*** **rs2605100 genotype.** Data are unadjusted means ± standard deviation or medians (interquartile range). Values of fasting serum triglycerides, serum insulin, plasma glucose, and HOMA-IR were logarithmically transformed prior to statistical analyses, and their effect sizes (β) are presented as the increase/decrease in percent. *P*-values were calculated assuming an additive model (*p*_add_). Interaction analysis (*p*_int_) was performed to test whether the effect of the alleles differed between men and women. All analyses were adjusted for age, sex, and BMI. Furthermore, waist circumference and waist-hip ratio were adjusted for age and sex, and the *p*-values are shown in parentheses.
(DOCX)

Table S3 ***LYPLAL1*** **rs2605100,** ***NRXN3*** **rs10146997,** ***MSRA*** **rs545854, and** ***TFAP2B*** **rs987237 in relation to central obesity.** Data are number of individuals, divided into genotype groups. The effect is either the odds ratio (OR) or the per allele effect size presented as the increase/decrease and 95%CI. Effect and *p*-values shown are for an additive genetic model (*p*_add_) and are adjusted for age, sex and diabetes treatment (without/ with BMI) for the obese cases, and QT analyses are adjusted for age and sex (without/with BMI). QT, quantitative trait; WC, waist circumference; WHR, waist-hip ratio.
(DOCX)

Table S4 ***LYPLAL1*** **rs2605100,** ***NRXN3*** **rs10146997,** ***MSRA*** **rs545854, and** ***TFAP2B*** **rs987237 in relation to general obesity.** Data are number of individuals, divided into genotype groups. The effect is either the odds ratio (OR) or the per allele effect size presented as the increase/decrease and 95%CI. Effect and *p*-values shown are for an additive genetic model (*p*_add_) and are adjusted for age, sex and diabetes treatment (without/ with WC) for the obese cases, and QT analyses are adjusted for age and sex (without/with WC). QT, quantitative trait.
(DOCX)

Table S5 **Quantitative metabolic traits in 5,789 treatment-naïve middle-aged Danes from the population-based Inter99 cohort according to** ***NRXN3*** **rs10146997 genotype.** Data are unadjusted means ± standard deviation or medians (interquartile range). Values of fasting serum triglycerides, serum insulin, plasma glucose, and HOMA-IR were logarithmically transformed prior to statistical analyses, and their effect sizes (β) are presented as the increase/decrease in percent. *P*-values were calculated assuming an additive model (*p*_add_). Interaction analysis (*p*_int_) was performed to test whether the effect of the alleles differed between men and women. All analyses were adjusted for age, sex, and BMI. Furthermore, waist circumference and waist-hip ratio were adjusted for age and sex, and the *p*-values are shown in parentheses.
(DOCX)

Table S6 **Quantitative metabolic traits in 5,804 treatment-naïve middle-aged Danes from the population-based Inter99 cohort according to** ***MSRA*** **rs545854 genotype.** Data are unadjusted means ± standard deviation or medians (interquartile range). Values of fasting serum triglycerides, serum insulin, plasma glucose, and HOMA-IR were logarithmically transformed prior to statistical analyses, and their effect sizes (β) are presented as the increase/decrease in percent. *P*-values were calculated assuming an additive model (*p*_add_). Interaction analysis (*p*_int_) was performed to test whether the effect of the alleles differed between men and women. All analyses were adjusted for age, sex, and BMI. Furthermore, waist circumference and waist-hip ratio were adjusted for age and sex, and the *p*-values are shown in parentheses.
(DOCX)

Table S7 **Quantitative metabolic traits in 5,764 treatment-naïve middle-aged Danes from the population-based Inter99 cohort according to** ***TFAP2B*** **rs987237 genotype.** Data are unadjusted means ± standard deviation or medians (interquartile range). Values of fasting serum triglycerides, serum insulin, plasma glucose, and HOMA-IR were logarithmically transformed prior to statistical analyses, and their effect sizes (β) are presented as the increase/decrease in percent. *P*-values were calculated assuming an additive model (*p*_add_). Interaction analysis (*p*_int_) was performed to test whether the effect of the alleles differed between men and women. All analyses were adjusted for age, sex, and BMI. Furthermore, waist circumference and waist-hip ratio were adjusted for age and sex, and the *p*-values are shown in parentheses.
(DOCX)

Table S8 **Statistical power estimates.** *Central overweight:* ∼4,500 controls and ∼3,700 overweight individuals. Prevalence = 0.23 (Men with waist circumference ≥94 cm and <102 cm and women with waist circumference ≥80 cm and <88 cm from the Inter99). *General overweight:* ∼3,200 controls and ∼7,000 overweight individuals. Prevalence = 0.39 (Individuals with BMI≥25 kg/m^2^ and BMI<30 kg/m^2^ from the Inter99). *Central obesity:* ∼4,500 controls and ∼7,000 obese individuals. Prevalence = 0.22 (men with waist circumference ≥102 cm and women with waist circumference ≥88cm from the Inter99). *General obesity:* ∼3,200 controls and ∼4,800 obese individuals. Prevalence = 0.17 (Individuals with BMI≥30 kg/m^2^ from the Inter99). *Type 2 diabetes:* ∼ 4,900 controls and ∼ 3,500 type 2 diabetics. Prevalence = 0.05 (Individuals with screen detected or known type 2 diabetes from the Inter99). The statistical power calculations for quantitative traits were estimated in R using 1,000 simulations and a significance threshold of 0.05. The statistical power calculations in the case-control analyses were done using CaTS, power calculations for large genetic association studies, available at http://www.sph.umich.edu/csg/abecasis/cats/. RAF  =  risk allele frequency.
(DOCX)
