# Supplementary material for: Implications of Central Obesity-Related Variants in LYPLAL1, NRXN3, MSRA, and TFAP2B on Quantitative Metabolic Traits in Adult Danes
Source: PLoS One. 2011 Jun 2;6(6):e20640. doi: 10.1371/journal.pone.0020640 (PMC3107232; doi:10.1371/journal.pone.0020640)
Supplement: Table S1 — Characteristics for individuals included in the analyses stratified according to study group. Data are means ± standard deviation. SDC, Steno diabetes center. WC, waist circumference. (DOCX) [file pone.0020640.s001.docx]

**Supplementary table 1**

|  | **The population-based Inter99 study sample** | **The SDC type 2 diabetic patients** | **The SDC population-based study group** | **The ADDITION study cohort** |
| --- | --- | --- | --- | --- |
| *n* [men/women] | 6,162  [3,070/3,092] | 1,695  [1,045/650] | 730  [332/398] | 6,739  [3,862/2,877] |
| Age (years)  [men/women] | 46.2±7.9  [46.6±7.8/45.9±8.0] | 62.4±11.3  [61.3±10.8/64.3±11.9] | 57.8±8.6  [58.9±8.4/56.9±8.6] | 59.7±6.8  [59.4±6.8/60.2±6.8] |
| Weight (kg)  [men/women] | 78.3±16.1  [85.7±14.1/70.9±14.3] | 89.7±18.9  [95.0±17.7/81.5±17.8] | 74.5±13.5  [81.8±12.0/68.4±11.5] | 88.3±14.3  [92.6±13.2/82.6±13.1] |
| Height (cm)  [men/women] | 172.3±9.2  [178.7±6.9/165.9±6.3] | 171.7±9.9  [177.5±7.0/162.7±6.5] | 169.3±9.0  [176.1±6.7/163.7±6.4] | 170.9±9.2  [176.5±6.6/163.3±6.2] |
| WC (cm)  [men/women] | 86.7±13.3  [93.2±11.0/80.3±12.2] | 104.8±14.8  [107.6±13.7/100.4±15.5] | 86.9±11.5  [93.4±9.3/81.6±10.3] | 101.2±11.5  [104.4±10.4/96.8±11.6] |
| BMI (kg/m^2^)  [men/women] | 26.3±4.6  [26.8±4.0/25.8±5.0] | 30.3±5.6  [30.1±5.1/30.8±6.3] | 25.9±3.8  [26.4±3.3/25.5±4.2] | 30.2±4.2  [29.7±3.7/30.9±4.7] |
