# Supplementary material for: Implications of Central Obesity-Related Variants in LYPLAL1, NRXN3, MSRA, and TFAP2B on Quantitative Metabolic Traits in Adult Danes
Source: PLoS One. 2011 Jun 2;6(6):e20640. doi: 10.1371/journal.pone.0020640 (PMC3107232; doi:10.1371/journal.pone.0020640)
Supplement: Table S2 — Quantitative metabolic traits in 5,769 treatment-naïve middle-aged Danes from the population-based Inter99 cohort according to LYPLAL1 rs2605100 genotype. Data are unadjusted means ± standard deviation or medians (interquartile range). Values of fasting serum triglycerides, serum insulin, plasma glucose, and HOMA-IR were logarithmically transformed prior to statistical analyses, and their effect sizes (β) are presented as the increase/decrease in percent. P-values were calculated assuming an additive model (p add). Interaction analysis (p int) was performed to test whether the effect of the alleles differed between men and women. All analyses were adjusted for age, sex, and BMI. Furthermore, waist circumference and waist-hip ratio were adjusted for age and sex, and the p-values are shown in parentheses. (DOCX) [file pone.0020640.s002.docx]

**Supplementary table 2**

| ***LYPLAL1***  **rs2605100** | **AA**  **(HO)** | **GA**  **(HE)** | **GG**  **(WT)** | **β (95% CI)** | ***p*_add_** | ***p*_int_** |
| --- | --- | --- | --- | --- | --- | --- |
| *n* (all) | 505 | 2458 | 2806 |  |  |  |
| *n* (men) | 265 | 1190 | 1414 |  |  |  |
| *n* (women) | 240 | 1268 | 1392 |  |  |  |
| Age (all), years | 46.6±7.9 | 46.1±7.9 | 46.1±7.9 |  |  |  |
| *BMI (kg/m^2^)* | | | | | | |
| All | 26.7+/-5.1 | 26.2+/-4.5 | 26.1+/-4.4 | -0.18 (-0.36;-1x10^-3^) | 0.05 | 0.81 |
| Men | 27.1+/-4.4 | 26.7+/-4 | 26.7+/-3.9 | -0.13 (-0.36;0.09) | 0.25 |  |
| Women | 26.2+/-5.8 | 25.6+/-4.8 | 25.6+/-4.8 | -0.23 (-0.51;0.05) | 0.10 |  |
| *Waist circumference (cm)* | | | | | | |
| All | 87.6+/-14.3 | 86.4+/-13.1 | 86.2+/-13.0 | -0.08 (-0.29;0.13) | 0.47 (0.04) | 0.53 (0.96) |
| Men | 93.8+/-12.4 | 93.1+/-11 | 92.6+/-10.7 | -0.15 (-0.43;0.13) | 0.30 (0.13) |  |
| Women | 80.8+/-13.1 | 80+/-11.7 | 79.8+/-11.8 | 0.02 (-0.29;0.33) | 0.90 (0.18) |  |
| *Waist-hip ratio* | | | | | | |
| All | 0.9+/-0.1 | 0.9+/-0.1 | 0.9+/-0.1 | -5x10^-4^ (-3x10^-3^;2x10^-3^) | 0.64 (0.19) | 0.35 (0.48) |
| Men | 0.9+/-0.1 | 0.9+/-0.1 | 0.9+/-0.1 | -3x10^-4^ (-3x10^-3^;3x10^-3^) | 0.84 (0.41) |  |
| Women | 0.8+/-0.1 | 0.8+/-0.1 | 0.8+/-0.1 | -4x10^-4^ (-4x10^-3^;3x10^-3^) | 0.80 (0.41) |  |
| *Fasting serum triglycerides (mmol/l)* | | | | | | |
| All | 1.1 (0.8-1.6) | 1.0 (0.8-1.5) | 1.1 (0.8-1.5) | 3 % (1;5) | 2.7 x 10^-3^ | 0.02 |
| Men | 1.2 (0.8-1.6) | 1.2 (0.8-1.7) | 1.2 (0.9-1.8) | 6 % (3;9) | 2.4 x 10^-4^ |  |
| Women | 1.0 (0.7-1.4) | 0.9 (0.7-1.3) | 1.0 (0.7-1.3) | 0.1 % (-2.4;2.6) | 0.94 |  |
| *Fasting serum insulin (pmol/l)* | | | | | | |
| All | 33 (24-52) | 33 (23-49) | 35 (24-52) | 3 % (1;5) | 2.5 x 10^-3^ | 0.09 |
| Men | 34 (23-50) | 36 (24-55) | 37 (25-56) | 3 % (-0.1;6) | 0.06 |  |
| Women | 32 (24-54) | 31 (23-45) | 33 (23-49) | 4 % (1;6) | 0.01 |  |
| *Fasting plasma glucose (mmol/l)* | | | | | | |
| All | 5.5 (5.1-5.9) | 5.4 (5.1-5.8) | 5.5 (5.1-5.8) | 0.3 % (-0.1;0.8) | 0.11 | 0.54 |
| Men | 5.6 (5.3-6.0) | 5.6 (5.3-6.0) | 5.6 (5.3-6.0) | 0.1 % (-0.5;0.8) | 0.69 |  |
| Women | 5.2 (5.0-5.6) | 5.3 (5.0-5.6) | 5.3 (5.0-5.6) | 1 % (0;1) | 0.04 |  |
| *Insulin resistance, HOMA-IR* | | | | | | |
| All | 8.0 (5.7-12.7) | 8.1 (5.5-12.4) | 8.6 (5.7-13.2) | 4 % (1;6) | 1.5 x 10^-3^ | 0.10 |
| Men | 9.0 (5.8-13.0) | 9.1 (6.0-14.2) | 9.2 (6.0-14.4) | 3 % (-0.2;6) | 0.07 |  |
| Women | 7.6 (5.4-12.4) | 7.5 (5.2-10.8) | 8.0 (5.3-11.9) | 4 % (1;7) | 6.0 x 10^-3^ |  |
