# Supplementary material for: Implications of Central Obesity-Related Variants in LYPLAL1, NRXN3, MSRA, and TFAP2B on Quantitative Metabolic Traits in Adult Danes
Source: PLoS One. 2011 Jun 2;6(6):e20640. doi: 10.1371/journal.pone.0020640 (PMC3107232; doi:10.1371/journal.pone.0020640)
Supplement: Table S3 — LYPLAL1 rs2605100, NRXN3 rs10146997, MSRA rs545854, and TFAP2B rs987237 in relation to central obesity. Data are number of individuals, divided into genotype groups. The effect is either the odds ratio (OR) or the per allele effect size presented as the increase/decrease and 95%CI. Effect and p-values shown are for an additive genetic model (p add) and are adjusted for age, sex and diabetes treatment (without/ with BMI) for the obese cases, and QT analyses are adjusted for age and sex (without/with BMI). QT, quantitative trait; WC, waist circumference; WHR, waist-hip ratio. (DOCX) [file pone.0020640.s003.docx]

**Supplementary table 3**

| ***LYPLAL1* rs2605100** | ***n*** | **Genotype distribution**  ***n* AA/GA/GG** | **Effect**  **(95% CI)** | ***p*_add_** | ***p*_int_** | ***Heterogeneity***  ***(I^2^, p)*** |
| --- | --- | --- | --- | --- | --- | --- |
| **Controls** |  |  |  |  |  |  |
| **All** | 4350 | 373/1831/2146 |  |  |  |  |
| **Men** | 2412 | 207/992/1213 |  |  |  |  |
| **Women** | 1938 | 166/839/933 |  |  |  |  |
| **Obese case** |  |  |  |  | 0.37/0.13 |  |
| **All** | 6949 | 673/2979/3297 | 0.92(0.86-0.98) | 0.01/0.004 |  |  |
| **Men** | 3420 | 336/1491/1593 | 0.89(0.82-0.97) | 0.01/0.0004 |  |  |
| **Women** | 3529 | 337/1488/1704 | 0.94(0.86-1.04) | 0.25/0.83 |  |  |
| **QT – WC (cm)** |  |  |  |  | 0.06/0.15 | 0% [0%; 56%], 0.27 |
| **All** | 13011 | 1213/5573/6225 | -0.48 (-0.81;0.16) | 0.004/0.12 |  |  |
| **Men** | 6973 | 647/2988/3338 | -0.58 (-0.99;0.17) | 0.005/0.04 |  |  |
| **Women** | 6038 | 566/2585/2887 | -0.39 (-0.90;0.13) | 0.14/0.91 |  |  |
| **QT – WHR** |  |  |  |  | 0.44/0.40 | 0% [0%; 81%], 0.98 |
| **All** | 13011 | 1213/5573/6225 | -1x10^-3^ (-4x10^-3^;1x10^-3^) | 0.36/0.86 |  |  |
| **Men** | 6973 | 647/2988/3338 | -1x10^-3^ (-4x10^-3^;2x10^-3^) | 0.52/0.88 |  |  |
| **Women** | 6038 | 566/2585/2887 | -1x10^-3^ (-5x10^-3^;3x10^-3^) | 0.59/1.00 |  |  |
| ***NRXN3 r*s10146997** | ***n*** | **Genotype distribution**  ***n* AA/GA/GG** | **Effect**  **(95% CI)** | ***p*_add_** | ***p*_int_** |  |
| **Controls** |  |  |  |  |  |  |
| **All** | 4332 | 2711/1430/191 |  |  |  |  |
| **Men** | 2332 | 1477/811/106 |  |  |  |  |
| **Women** | 1938 | 1234/619/85 |  |  |  |  |
| **Obese cases** |  |  |  |  | 0.35/0.43 |  |
| **All** | 6757 | 4062/2374/321 | 1.06(0.99-1.14) | 0.11/0.31 |  |  |
| **Men** | 3310 | 1982/1188/140 | 1.03 (0.93-1.14) | 0.53/0.52 |  |  |
| **Women** | 3447 | 2080/1186/181 | 1.09(0.98-1.22) | 0.11/0.37 |  |  |
| **QT – WC** |  |  |  |  | 0.36/0.32 | 37% [0%; 78%], 0.80 (fixed) |
| **All** | 12728 | 7845/4292/591 | 0.15 (-0.22;0.52) | 0.43/0.58 |  |  |
| **Men** | 6796 | 4187/2295/314 | -0.10 (-0.57;0.37) | 0.67/0.12 |  |  |
| **Women** | 5932 | 3658/1997/277 | 0.40 (-0.18;0.98) | 0.17/0.44 |  |  |
| **QT – WHR** |  |  |  |  | 0.15/0.09 | 0% [0%; 70%], 0.53 |
| **All** | 12728 | 7845/4292/591 | 1x10^-3^ (-2x10^-3^;4x10^-3^) | 0.44/0.75 |  |  |
| **Men** | 6796 | 4187/2295/314 | -1x10^-3^ (-5x10^-3^;2x10^-3^) | 0.40/0.10 |  |  |
| **Women** | 5932 | 3658/1997/277 | 4x10^-3^ (-2x10^-4^;8x10^-3^) | 0.07/0.08 |  |  |
| ***MSRA* rs545854** | ***n*** | **Genotype distribution**  ***n* CC/GC/GG** | **Effect**  **(95% CI)** | ***p*_add_** | ***p*_int_** |  |
| **Controls** |  |  |  |  |  |  |
| **All** | 4364 | 3133/1131/100 |  |  |  |  |
| **Men** | 2427 | 1748/616/63 |  |  |  |  |
| **Women** | 1937 | 1385/515/37 |  |  |  |  |
| **Obese cases** |  |  |  |  | 0.07/0.19 |  |
| **All** | 6956 | 4889/1880/187 | 1.08(1.00-1.18) | 0.05/0.02 |  |  |
| **Men** | 3418 | 2396/942/80 | 1.08(0.97-1.21) | 0.17/0.22 |  |  |
| **Women** | 3538 | 2493/938/107 | 1.09(0.96-1.23) | 0.18/0.04 |  |  |
| **QT – WC** |  |  |  |  | 0.002/0.09 | 47% [0%; 82%], 0.91 (fixed) |
| **All** | 13031 | 9291/3404/336 | 0.16 (-0.25;0.56) | 0.46/0.85 |  |  |
| **Men** | 6974 | 4987/1815/172 | 0.25 (-0.27;0.77) | 0.35/0.50 |  |  |
| **Women** | 6057 | 4304/1589/164 | 0.05 (-0.60;0.69) | 0.89/0.37 |  |  |
| **QT – WHR** |  |  |  |  | 0.22/0.34 | 45% [0%; 84%], 0.74 |
| **All** | 13031 | 9291/3404/336 | 1x10^-3^ (-2x10^-3^;4x10^-3^) | 0.38/0.49 |  |  |
| **Men** | 6974 | 4987/1815/172 | 3x10^-3^ (-1x10^-3^;7x10^-3^) | 0.18/0.13 |  |  |
| **Women** | 6057 | 4304/1589/164 | 1x10^-4^ (-5x10^-3^;4x10^-3^) | 0.96/0.76 |  |  |
| ***TFAP2B* rs987237** | ***n*** | **Genotype distribution**  ***n* AA/GA/GG** | **Effect**  **(95% CI)** | ***p*_add_** | ***p*_int_** |  |
| **Controls** |  |  |  |  |  |  |
| **All** | 4341 | 3011/1217/113 |  |  |  |  |
| **Men** | 2400 | 1643/685/72 |  |  |  |  |
| **Women** | 1941 | 1368/532/41 |  |  |  |  |
| **Obese cases** |  |  |  |  | 0.04/0.0002 |  |
| **All** | 6921 | 4681/2022/218 | 1.08 (1.00-1.17) | 0.06/0.81 |  |  |
| **Men** | 3404 | 2322/983/99 | 1.01 (0.91-1.13) | 0.83/0.01 |  |  |
| **Women** | 3517 | 2359/1039/119 | 1.17 (1.03-1.32) | 0.01/0.001 |  |  |
| **QT – WC** |  |  |  |  | 0.10/0.13 | 50% [0%; 84%], 0.97 |
| **All** | 12956 | 8899/3697/360 | 0.44 (0.04;0.84) | 0.03/0.96 |  |  |
| **Men** | 6934 | 4758/1987/189 | 0.18 (-0.33;0.68) | 0.49/0.19 |  |  |
| **Women** | 6022 | 4141/1710/171 | 0.73 (0.10;1.36) | 0.02/0.20 |  |  |
| **QT – WHR** |  |  |  |  | 0.80/0.98 | 0% [0%; 87%], 0.46 |
| **All** | 12956 | 8899/3697/360 | -1x10^-3^ (-4x10^-3^;2x10^-3^) | 0.65/0.40 |  |  |
| **Men** | 6934 | 4758/1987/189 | -1x10^-3^ (-5x10^-3^;3x10^-3^) | 0.64/0.53 |  |  |
| **Women** | 6022 | 4141/1710/171 | -2x10^-4^ (-5x10^-3^;4x10^-3^) | 0.91/0.66 |  |  |
