# Supplementary material for: Implications of Central Obesity-Related Variants in LYPLAL1, NRXN3, MSRA, and TFAP2B on Quantitative Metabolic Traits in Adult Danes
Source: PLoS One. 2011 Jun 2;6(6):e20640. doi: 10.1371/journal.pone.0020640 (PMC3107232; doi:10.1371/journal.pone.0020640)
Supplement: Table S8 — Statistical power estimates. Central overweight: ∼4,500 controls and ∼3,700 overweight individuals. Prevalence = 0.23 (Men with waist circumference ≥94 cm and <102 cm and women with waist circumference ≥80 cm and <88 cm from the Inter99). General overweight: ∼3,200 controls and ∼7,000 overweight individuals. Prevalence = 0.39 (Individuals with BMI≥25 kg/m2 and BMI<30 kg/m2 from the Inter99). Central obesity: ∼4,500 controls and ∼7,000 obese individuals. Prevalence = 0.22 (men with waist circumference ≥102 cm and women with waist circumference ≥88cm from the Inter99). General obesity: ∼3,200 controls and ∼4,800 obese individuals. Prevalence = 0.17 (Individuals with BMI≥30 kg/m2 from the Inter99). Type 2 diabetes: ∼ 4,900 controls and ∼ 3,500 type 2 diabetics. Prevalence = 0.05 (Individuals with screen detected or known type 2 diabetes from the Inter99). The statistical power calculations for quantitative traits were estimated in R using 1,000 simulations and a significance threshold of 0.05. The statistical power calculations in the case-control analyses were done using CaTS, power calculations for large genetic association studies, available at http://www.sph.umich.edu/csg/abecasis/cats/. RAF = risk allele frequency. (DOCX) [file pone.0020640.s008.docx]

**Supplementary table 8**

|  |  | Power | | |
| --- | --- | --- | --- | --- |
|  | RAF | 15 | 20 | 70 |
| **Quantitative trait analyses** | 0.35 change in BMI units | 85% | 93% | 97% |
|  | 0.85 cm change in waist circumference | 72% | 80% | 90% |
|  | 0.007 change in waist-hip ratio | 83% | 91% | 97% |
|  | 5% change in fasting serum triglyceride | 95% | 98% | 99% |
|  | 1% change in fasting plasma glucose | 91% | 95% | 98% |
|  | 5% change in fasting serum insulin | 89% | 93% | 97% |
| **Case-control analyses** |  |  |  |  |
| Central overweight | OR of 1.10 | 81% | 88% | 92% |
|  | OR of 1.15 | 99% | 100% |  |
| General overweight | OR of 1.10 | 96% | 99% | 99% |
|  | OR of 1.15 | 100% |  |  |
| Central obesity | OR of 1.10 | 90% | 95% | 97% |
|  | OR of 1.15 | 100% |  |  |
| General obesity | OR of 1.10 | 73% | 81% | 86% |
|  | OR of 1.15 | 96% | 99% | 99% |
|  | OR of 1.20 | 100% |  |  |
| Type 2 diabetes | OR of 1.10 | 63% | 72% | 78% |
|  | OR of 1.15 | 92% | 96% | 97% |
|  | OR of 1.20 | 99% | 100% |  |
